# Antibiotic prescribing patterns for coronavirus disease 2019 (COVID-19) in two emergency departments with rapid procalcitonin

**DOI:** 10.1017/ice.2020.1329

**Published:** 2020-11-19

**Authors:** Michael S. Pulia, Ian Wolf, Rebecca J. Schwei, Derrick Chen, Alexander J. Lepak, Lucas T Schulz, Nasia Safdar

**Affiliations:** 1 BerbeeWalsh Department of Emergency Medicine University of Wisconsin-Madison School of Medicine and Public Health, Madison, Wisconsin; 2 University of Wisconsin Madison School of Medicine and Public Health, Madison, Wisconsin; 3 Department of Pathology and Laboratory Medicine, University of Wisconsin–Madison, Madison, Wisconsin; 4 Department of Medicine Division of Infectious Disease, University of Wisconsin Madison School of Medicine and Public Health, Madison, Wisconsin; 5 Department of Pharmacy, University of Wisconsin Health, Madison, Wisconsin

As the coronavirus disease 2019 (COVID-19) pandemic continues to spread, discussion around the appropriate use of antibiotics in these patients has evolved. Initially, treatment guidelines for COVID-19 recommended empiric antibiotic usage, especially among those with severe disease.^1^ However, a systematic review indicated that, on average, 72% of COVID-19 patients received antibiotic therapy yet only 8% of patients had microbiologically confirmed coinfections.^2^ Currently, the World Health Organization (WHO) recommends antibiotics only for patients with moderate COVID-19 and a suspected bacterial infection.^3^

Although the available data indicate overuse of antibiotics in COVID-19 patients, available reports lack sufficient detail (eg, timing of administration and clinical scenario) to properly evaluate appropriateness. In response to this gap in the literature, we conducted a detailed analysis of antibiotic prescribing patterns for a cohort of emergency department (ED) patients confirmed to have symptomatic COVID-19. Our primary objectives were to improve characterization of ED-based antibiotic prescribing and to assess the real-world effectiveness of procalcitonin (PCT) testing as a stewardship intervention during the COVID-19 pandemic.

## Methods

### Study design, setting and selection of participants

We conducted a retrospective cohort study using data from the electronic health record. All symptomatic patients who tested positive for COVID-19 at 2 Midwestern EDs in the same healthcare system between March 15, 2020, and May 18, 2020, were included. The institutional review board approved this study.

### Laboratory testing

COVID-19 testing was performed using nucleic acid amplification tests (NAATs) approved by the FDA for the detection of SARS-CoV-2 RNA in nasopharyngeal specimens. Our institution has a clinical guideline for the use of PCT testing in antibiotic decision-making for respiratory infections based on published cutoff values.^4^

### Collection of clinical and laboratory data

To characterize ED antibiotic prescription patterns, patients were first categorized as having received antibiotics (yes/no). Antibiotics were classified by spectrum, provider-selected indication, and route of administration. We abstracted the time stamp of the antibiotic order and all laboratory tests. Basic demographic information, including gender, race, Hispanic/Latinx status, and age were captured along with disposition, comorbidities, symptoms, and month of test. We recorded the COVID-19 WHO clinical score of the patient at time of the ED encounter and their worst clinical score in the 30-day period.^5^

### Statistical analysis

We compared differences in abstracted variables between patients that received and did not receive antibiotics in the ED using a χ^2^ test or Fisher exact test as appropriate. The difference in antibiotic prescribing rates based on PCT utilization were reported using a 2-sample test of proportions. All statistical analysis was done using R statistical software (R Foundation for Statistical Computing, Vienna, Austria) with α of ≤.05 considered significant.

## Results

A consecutive cohort of 73 ED patients with NAAT-confirmed, symptomatic COVID-19 were included. Table 1 describes the characteristics of the patients in the sample. Overall, 27 patients (37.0%) were prescribed antibiotics during their ED encounter. Of these patients, 25 (92.6%) received their antibiotics prior to their positive COVID-19 test result. Overall, 24 patients (88.8%) had antibiotics administered in the ED and 3 (11.1%) received antibiotics at discharge from the ED. In total, 47 unique antibiotic prescriptions were identified, with pneumonia being the most common indication listed (52.1%) followed by sepsis or bacteremia (27.1%) and urinary tract infection (12.5%). A PCT test was ordered for 45 patients (61.6%) during their ED encounter, with 10 patients (22.2%) having an elevated level (>0.25 µg/L). Of the 32 patients who had a PCT result available prior to an antibiotic order being placed, 25.0% received antibiotics as compared to 46.3% of those who either had no PCT testing done or the result was only available after the antibiotic order (−21.3%; 95% CI, −42.74% to −0.06%; *P =* .061).

**Table 1.** Patient Demographic and Clinical Encounter Characteristics Overall and by Antibiotic Group

Characteristic	Overall (n=73), No. (%)	No Antibiotic (n=46), No. (%)	Antibiotic (n=27), No. (%)	*P* Value^a^
**Age, y**				.067
0–19	6 (8)	5 (11)	1 (4)	
20–49	21 (29)	16 (35)	5 (19)	
50–59	13 (18)	8 (17)	5 (19)	
60–69	10 (14)	4 (9)	6 (22)	
70–79	10 (14)	3 (7)	7 (26)	
80+	13 (18)	10 (22)	3 (11)	
Sex, female	38 (52)	25 (54)	13 (48)	.609
Hispanic or Latinx	13 (18)	9 (20)	4 (15)	.756
**Race**				.577
White	51 (70)	30 (65)	21 (78)	
Black	18 (25)	13 (28)	5 (19)	
Other	4 (6)	3 (7)	1 (4)	
**Medical history**				
Diabetes	22 (30)	13 (28)	9 (33)	.648
Hypertension	38 (52)	25 (54)	13 (48)	.609
Heart disease	10 (14)	9 (20)	1 (4)	.080
COPD	4 (6)	3 (7)	1 (4)	1.00
Asthma	10 (14)	6 (13)	4 (15)	1.00
Other lung disease^b^	3 (4)	1 (2)	2 (7)	.551
**Symptoms**				
Respiratory	61 (84)	39 (85)	22 (82)	.751
Fever	51 (70)	32 (70)	19 (70)	1.00
GI	37 (51)	24 (52)	13 (48)	.740
**Date of COVID test**				.522
March	29 (40)	16 (35)	13 (48)	
April	26 (36)	18 (39)	8 (30)	
May	18 (25)	12 (26)	6 (22)	
**Clinical score at ED visit/test**				.461
1–3	28 (38)	16 (35)	12 (44)	
4+	45 (62)	30 (65)	15 (56)	
**Disposition**				.185
Discharged	26 (36)	19 (41)	7 (26)	
Admitted	47 (64)	27 (59)	20 (74)	
Mortality within 30 d	9 (12)	5 (11)	4 (15)	.718
Intubation/mechanical ventilation	8 (11)	4 (9)	4 (15)	.457
**Procalcitonin**				.029
≤0.25	35 (78)	22 (92)	13 (62)	
>0.25	10 (22)	2 (8)	8 (38)	

Note. COPD, chronic obstructive pulmonary disease; GI, gastrointestinal; ED, emergency department.

a Comparison of group that had antibiotics prescribed and group that did not have antibiotics prescribed.

b Other lung disease includes pulmonary fibrosis, cystic fibrosis, bronchiectasis, and pulmonary hypertension.

## Discussion

Widespread use of antibiotics for COVID-19 has been reported worldwide, and the resulting risk of increased bacterial resistance is increasingly recognized as a parallel public health crisis to the ongoing pandemic.^6^ The low rate of observed bacterial coinfection rates among COVID-19 patients suggests that there is an opportunity to safely avoid routine, empiric prescribing of antibiotics for this population in the ED. We present the first detailed analysis, including event timing of PCT utilization and antibiotic prescribing, among patients diagnosed with symptomatic COVID-19 in the ED.

The overall prescribing rate in our 2 EDs (37%) is at the lower end of the reported ranges for overall COVID-19 antibiotic use.^7^ One potential explanation for the low overall prescribing rates despite long turnaround times for COVID-19 test results is the utilization of rapid PCT to guide empiric antibiotic decision. There was a −21.3% absolute difference in antibiotic prescribing for patients who received PCT testing. Supporting this interpretation, during our detailed chart abstractions, we came across numerous quotations indicating how PCT factored into this decision making, such as “Antibiotics held as PCT negative despite 9 days of symptoms—likely viral etiology.” Notably, most PCT results were negative, even among confirmed COVID-19 patients with a higher acuity level, as indicated by the majority having WHO scores ≥4 (ie, requiring supplemental oxygen). This finding is consistent with previous studies reporting negative PCT values for the majority of COVID-19 patients.^8–10^

COVID-19 has presented a significant challenge to antibiotic stewardship. Our observations suggest that it is possible, after excluding patients with sepsis or identified nonpulmonary infections (eg, UTI), to reduce empiric ED prescribing rates so they more closely align with observed bacterial coinfection rates. Interventional studies examining the role of biomarkers and rapid diagnostics are urgently needed to identify effective stewardship strategies for ED patients with suspected or confirmed COVID-19.
